# Early gene expression profiling through a macro-array approach on non-apoptotic human monocytes fed with hemozoin (malarial pigment)

**DOI:** 10.1186/1475-2875-9-S2-P39

**Published:** 2010-10-20

**Authors:** Mauro Prato, Daniela Ulliers, Valentina Gallo, Evelin Schwarzer, Oscar B Akide-Ndunge, Giorgia Mandili, Amina Khadjavi, Silvia Saviozzi, Raffaele A Calogero, Paolo Arese, Giuliana Giribaldi

**Affiliations:** 1 Department of Genetics, Biology and Biochemistry, University of Torino, Torino, Italy; 2 Department of Clinical and Biological Sciences, University of Torino, San Luigi Hospital, Orbassano, Torino, Italy

## Background

Hemozoin-fed (HZ-fed) monocytes are strongly exposed to oxidative stress, shed large amounts of peroxidation derivatives with subsequent impairment of numerous functions and overproduce proinflammatory cytokines. Nevertheless histopathologic features of autoptic tissues from patients with severe malaria show abundant presence of HZ in Kupffer cells and other tissue macrophages, suggesting that function impairment and cytokines production are not accompanied by cells death. The aim of present study is to clarify the role of hemozoin on cell survival focusing on the involvement and temporal setting of proinflammatory and anti-apoptotic molecules.

## Materials and methods

Short term gene expression analysis was performed through macro-array of a complete panel of cytokines and confirming real-time RT-PCR. Intermediate term HSPs protein expression was evaluated by western blotting. Long term cell viability was analysed by immunocytochemistry and flow cytometry.

## Results

Short term gene expression analysis (0-4h after 2h-phagocytosis) showed that HZ induced immediately IL-1 β gene expression, furtherfollowed by additional transcription of eight chemokines (IL-8, ENA-78, GROα, GROβ, GROγ, MIP-1α, MIP-1β and MCP-1), two cytokines (TNFα and IL-1RA), and cytokine/chemokine-related proteolytic enzyme MMP-9. Furthermore, real-time RT-PCR analysis showed that 15-HETE, a potent lipoperoxidation derivative generated by HZ through heme-catalysis, recapitulated HZ effects on five chemokines expression. Intermediate term investigation (9h after 2h-phagocytosis) showed that HZ increased protein expression of HSP27, a chemokine-related molecule with anti-apoptotic properties. Finally, long term cell viability (24-72h after 2h-phagocytosis) was unaffected by HZ in human monocytes.

## Conclusions

Collectively, present data suggest that apoptosis of HZ-fed monocytes is prevented through a cascade involving 15-HETE-mediated higher transcription of IL-1 β, rapidly endorsed by chemokines, TNFα, MMP-9 and IL-1RA transcription and up-regulation of anti-apoptotic HSP27 protein expression, favouring persistence of impaired monocytes in the bloodstream and clinical progress towards fatal complicated malaria.

